# Spincare System Demonstrates Safety and Efficacy in Treating Partial-Thickness Burns

**DOI:** 10.1093/jbcr/irae024

**Published:** 2024-02-21

**Authors:** Josef Haik, Yehuda Ullmann, Eyal Gur, Erik Biros, Rachel Kornhaber, Michelle Cleary, Dani Kruchevsky, Sivan Zissman, Yossi Namir, Moti Harats

**Affiliations:** Department of Plastic and Reconstructive Surgery, Sheba Medical Center, Ramat-Gan, affiliated with the School of Medicine, Tel-Aviv University, Tel-Aviv, 52621, Israel; Talpiot Leadership Program, Sheba Medical Center, Tel-Hashomer, Israel; Institute for Health Research, University of Notre Dame, Fremantle, 6160, WA, Australia; Department of Plastic and Reconstructive Surgery, Rambam Health Care Campus, affiliated with the Rappaport Faculty of Medicine, Technion, Haifa, 3109601, Israel; Department of Plastic and Reconstructive Surgery, Sourasky Medical Center, affiliated with the School of Medicine, Tel-Aviv University, Tel-Aviv, 6423906, Israel; College of Medicine and Dentistry, James Cook University, Townsville, 4810, Australia; Townville University Hospital, Townsville,4810, Australia; Department of Plastic and Reconstructive Surgery, Sheba Medical Center, Ramat-Gan, affiliated with the School of Medicine, Tel-Aviv University, Tel-Aviv, 52621, Israel; School of Nursing, Paramedicine and Healthcare Sciences, Charles Sturt University, Bathurst, NSW, 2795, Australia; School of Nursing, Midwifery and Social Sciences, CQUniversity, Sydeney, NSW, 2000, Australia; Department of Plastic and Reconstructive Surgery, Rambam Health Care Campus, affiliated with the Rappaport Faculty of Medicine, Technion, Haifa, 3109601, Israel; Department of Plastic and Reconstructive Surgery, Sourasky Medical Center, affiliated with the School of Medicine, Tel-Aviv University, Tel-Aviv, 6423906, Israel; Department of Plastic and Reconstructive Surgery, Sourasky Medical Center, affiliated with the School of Medicine, Tel-Aviv University, Tel-Aviv, 6423906, Israel; Department of Plastic and Reconstructive Surgery, Sheba Medical Center, Ramat-Gan, affiliated with the School of Medicine, Tel-Aviv University, Tel-Aviv, 52621, Israel; Talpiot Leadership Program, Sheba Medical Center, Tel-Hashomer, Israel; Institute for Health Research, University of Notre Dame, Fremantle, 6160, WA, Australia

**Keywords:** burns, partial-thickness, electrospinning

## Abstract

Partial-thickness burns are the most common form of burns, affecting the dermis and possibly resulting in scarring and infection. The Spincare System is a new device that uses electrospinning technology to create a temporary skin-like matrix that can be applied to wounds. This study evaluated the performance, safety, and efficacy of Spincare in treating superficial to partial-thickness burns not considered for surgery. A prospective single-arm, open-label, multicenter study was conducted in 3 adult burn units across Israel. Forty-four patients with superficial to intermediate burns of up to 10% of TBSA were enrolled. Spincare was applied to the wounds, and follow-up visits were performed on days 7, 14, and 21 and months 3 and 6 posttreatment. Thirty-one patients with 36 wounds completed the day 21 visit. The mean wound healing area on day 21 was 97.26 ± 9.41%, and the mean healing time was 12.8 ± 4.3 days. Only one moderate adverse event was observed concerning the treatment, and it is important to acknowledge the potential progression of this hypertrophic scar into a keloid. This study demonstrated that Spincare is a safe and effective device for treating superficial to intermediate partial-thickness burns. Spincare achieved rapid and complete wound healing with a low incidence of adverse events.

## INTRODUCTION

Burn wound healing involves a well-organized, complex process that leads to the repair of injured tissues.^1,2^ The severity of burn injuries dramatically varies, and morbidity and mortality tend to increase as the surface area of burns increases.^3^ Generally, burn wounds of partial-thickness that are appropriately managed show measurable progress and closure within 2–3 weeks. Current appropriate medical practice for full-thickness burns is the early excision and coverage with skin grafts or substitutes. For partial-thickness burns, burn clinicians are faced with whether to excise the burn or leave it for primary healing, having implications for scarring that represent a significant health burden and drain on resources, contributing to a substantial disability, morbidity, and cost.^4^

Device technology in wound care has progressed substantially over the recent decades, helping to revolutionize how clinicians approach wound management.^5^ As the industry continues to develop new technologies and refine existing ones, we can expect to see more innovative solutions. Burn care has progressed from labor-intensive painful dressings to intelligent dressings requiring less intervention.^6^ Today, device technology plays an increasingly important role in wound care, offering new and innovative ways to promote healing and prevent infection.^7^

One such technology is electrospinning, a novel technology that can treat wounds.^8^ The electrospun nanofibrous polymer-based matrix, which is applied to the wound bed, consists of a thin, flexible film that spun out of a solution of polymers. The electrospun solution creates, in situ, a uniform, steady stream of nanofibers that deposits on the wound. The electrospinning process creates a nanofibrous polymer-based matrix with properties ideal for wound care, mimicking the extracellular matrix structure, and providing temporary skin-like protecting coverage to the wound.^9^ Electrospun nanofibers have not only been preclinically tested using animal wound healing models^8^ but also have been used in donor sites and found to be safe and effective.^9^ Here, we evaluated the performance, safety, and efficacy of the Spincare System and matrix in managing superficial to intermediate partial-thickness burns.

## MATERIALS AND METHODS

### Study design

A prospective, single-arm, open-labeled multicenter study for treating partial-thickness burns (not considered for surgery) was performed to evaluate the effectiveness and safety of the Spincare System, representing a bedside electrospinning device that applies a nanofibrous matrix that mimics the structure of the extracellular matrix and thereby enhances fibroblast adhesion and proliferation (Spincare System, Nanomedic Technologies Ltd.).

The enrollment took place from March 2017 to August 2021. Recruitment was performed in 3 departments of plastic and reconstructive surgery in the following medical centers in Israel: Sheba Medical Center (Ramat-Gan), Rambam Health Care Campus (Haifa), and Sourasky Medical Center (Tel-Aviv).

The study included screening patients, 6 clinic visits, and unscheduled visits conducted as necessary. During the screening, patients were examined to determine their burn wound classification. All patients considered eligible for study participation received the Spincare matrix according to the Spincare instructions for use on the patient’s day 1 of the study. Follow-up visits were performed on days 7, 14, and 21 posttreatment, during which the matrix could be maintained, removed, or replaced as per the Principal Investigator’s (PI) decision. The extended follow-up visits were conducted 3 and 6 months posttreatment.

### Ethics

The study was conducted according to the good clinical practice (GCP) guidelines as described in the 21 Case Report Form 812.28(a)(1), ISO 14155:2020, and ICH GCP Guideline (amended in 2016) documents. Also, the Declaration of Helsinki (7th revision 2013) and all applicable regulatory requirements, including local requirements per the Israeli Ministry of Health, were followed. Furthermore, the Ethics Committee approvals for the study, the patient information sheet, and the consent form were required before the study began.

The Institutional Review Board of each contributing center approved the study protocol following the Helsinki Declaration, and written consent was obtained from each participant before participating in the study. The Israeli Ministry of Health also approved the current study. This trial was registered at ClinicalTrials.gov as NCT02997592 (https://clinicaltrials.gov/ct2/show/NCT02997592).

### Patients

To be included in this study, patients had to meet all the inclusion criteria and none of the exclusion criteria. The inclusion criteria included ≥18 years old of both sexes with partial-thickness burn wounds (superficial to intermediate) of up to 10% of the TBSA, of which the target wound for treatment is up to 5% TBSA or up to 300 cm^2^. In addition, patients had to present at the emergency room within the first 24–48 hours postinjury and have their wounds debrided. Finally, all patients had to give written informed consent before enrolling in the study.

Exclusion criteria included any known systemic infection, malignancy, major systemic disorder (hepatic, renal, neurologic, or endocrinologic disorders), dermatological disorders (psoriasis, pemphigus, Steven Johnson), psychiatric conditions, burns greater than specified above, full-thickness burns as well as pregnant or nursing females. In addition, patients with electrical or chemical burns were excluded. Finally, patients participating in another clinical trial within 30 days before the screening visit were excluded.

### Outcome measures

Our 2 primary outcomes were effectiveness and safety. The effectiveness was measured as wound healing and re-epithelialization up to day 21, where the burn was considered healed when at least 95% of the total area was re-epithelialized and no dried serous exudate was evident.

The number of healed wounds on day 21 was cumulative, that is, wounds that already healed up to day 21 were counted on day 21 even if complete healing (re-epithelialization ≥95%) was obtained on day 7 or day 14 or during an unscheduled visit performed up to day 21. The safety was measured using the Draize Dermal Irritation Scoring system (DDIS) described elsewhere.^10^

Secondary outcomes included several indices: pain assessment (using the Visual Analog Scale (VAS) scale at rest, during the Spincare matrix application, and postapplication), itching (using the VAS scale at 3 and 6 months), scarring (using the Vancouver Scar Scale at 3 and 6 months), percent of patients with infection, time of application (using numerical rating scale), and ease of use (using 0–5 numerical rating scale).

In addition, adverse events (AE) were coded using the Medical Dictionary for Regulatory Activities (MedDRA) Version 19.1 (or higher) terminology and presented in tables by System Organ Class (SOC) and Preferred Term (PT). The AE data were listed individually, and the frequency of device-related AE was summarized in tables by SOC, PT, and seriousness.

### Statistical analysis

All measured variables and derived parameters were listed individually and, if appropriate, tabulated by descriptive statistics. In addition, for categorical variables, summary tables were provided, giving sample size and absolute and relative frequency. Summary tables were provided for continuous variables, giving sample size, arithmetic mean, standard deviation, median, minimum, and maximum. All data were analyzed using SPSS Version 25 or Stata 16.

## RESULTS

### Patients

A total of 44 patients (52 wounds) were enrolled in the study (Table 1). In total, 31 (70.5%) patients completed the day 21 visit (primary end point), and 29 (65.9%) patients completed the day 21 visit and 3- and 6-month follow-ups (extended follow-ups). Of the 15 (34.1%) patients who withdrew early from the study, 8 (18.1%) patients were lost to follow-up, 4 (9.1%) patients withdrew their consent; the investigator withdrew 3 (6.8%) patients because their burns were deeper and met exclusion criteria.

**Table 1.** Patient Enrollment and Clinical Characteristics

Characteristic	Patients
Did the patient complete the follow-up visit on day 21?	
No	13 (29.5%)
Yes	31 (70.5%)
Did the patient complete the study for the month 6 visit?	
No	15 (34.1%)
Yes	29 (65.9%)
If no, specify the reason	
Lost to follow-up	8 (18.1%)
Withdraw consent	4 (9.1%)
Investigator withdraw patient	3 (6.8%)
Sex, *n* (%)	*N* = 44
Males	28 (63.6)
Females	16 (36.4)
Age, years	*N* = 44
Mean ± SD	39.98 ± 15.26
Median (min, max)	37.5 (18, 86)
Weight, kg	*N* = 37
Mean ± SD	72.45 ± 15.73
Median (min, max)	70.0 (42.0, 101.0)
Height, cm	*N* = 35
Mean ± SD	170.86 ± 8.85
Median (min, max)	168.0 (156.0, 189.0)
Total TBSA (%)	*N* = 44
Mean ± SD	6.25 ± 2.89
Median (min, max)	6.0 (1.0, 10.0)

Of the total cohort of 44 patients (Table 1), 28 (63.6%) patients were males, and 16 (36.4%) were females. The patients’ mean age was 39.98 ± 15.26 years, and the minimum and maximum age was 18 and 86 years, respectively. The subjects’ mean height and weight were 170.86 ± 8.85 cm and 72.45 ± 15.73 kg, respectively. The mean total TBSA (%) was 6.25 ± 2.89%.

### Primary outcome—effectiveness

Effectiveness was assessed as the number (percent, %) of wounds healed, where a wound was considered healed if re-epithelization was ≥95% of the total wound area. A cumulative number of 10/42 (23.8%), 31/38 (81.6%), and 35/38 (92.1%) wounds were considered healed up to days 7, 14, and 21, respectively (Table 2). The healing was subsequently examined at follow-up months 3 and 6 and found to be 38/38 (100%, of which 26 were intermediate wounds and 12 were superficial wounds) at both time points.

**Table 2.** Cumulative Percent of Healing Wounds^a^

Cumulative number of wounds healed (≥95%)^b^		All	
		N	%
Follow-up visit (day 7)	No	32	76.2
	Yes	10	23.8
Follow-up visit (day 14)	No	7	18.4
	Yes	31	81.6
Follow-up visit (day 21)	No	3	7.9
	Yes	35	92.1

^a^Note that cumulative numbers are taking in account wounds healed up to the respective follow-up visit. ^b^ Wound healing considered complete at ≥95% re-epithelization

**Figure 1.**
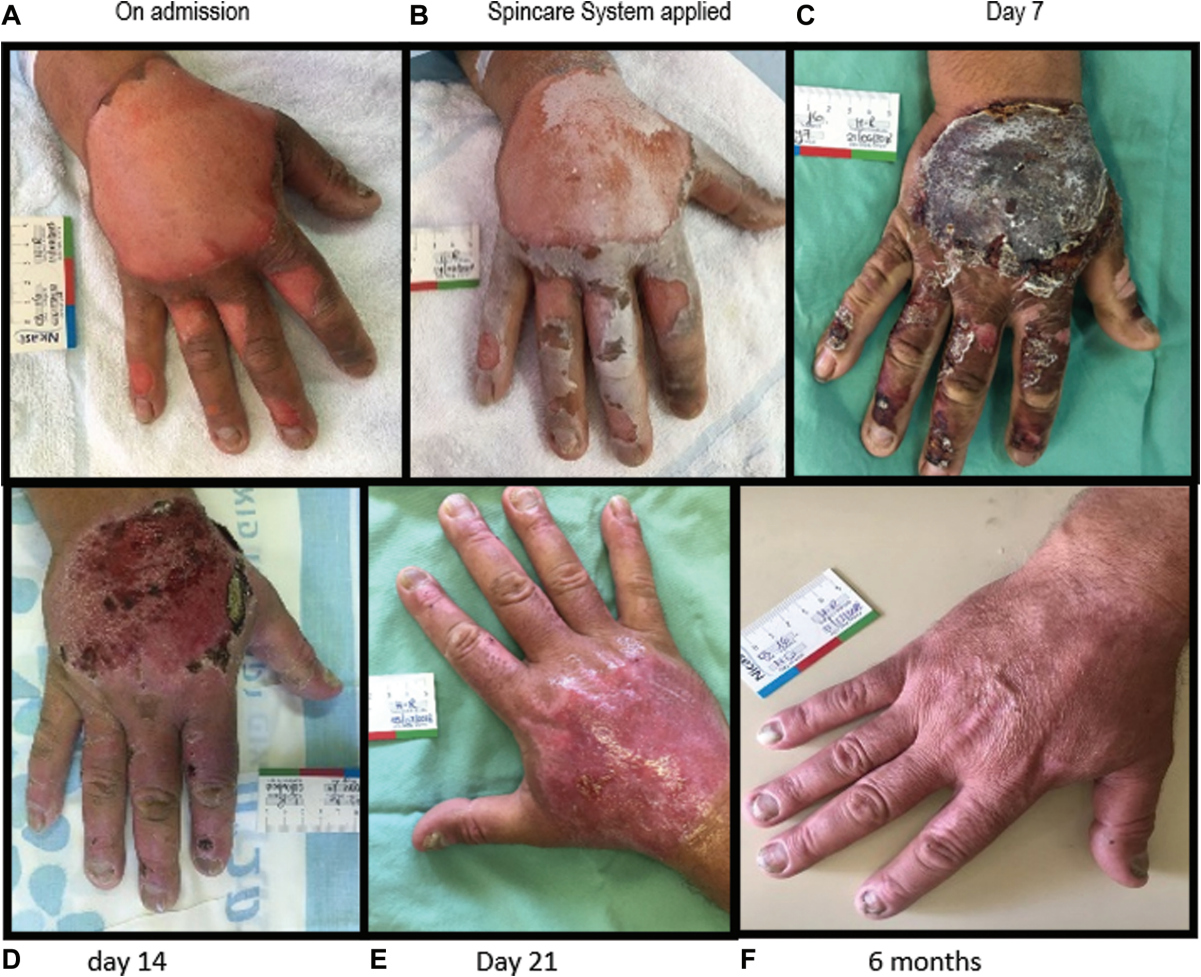
Fifty-Four-Year-Old Male, Right-Hand Superficial to Intermediate Partial-Thickness Burn (A) Treated With Spincare Matrix (B) 24 h Postinjury and Posthypochlorite Solution and Blister Debridement. Smooth Healing Process (C–E) and Complete Healing Achieved by Day 14 With No Infection or Other Complication. Excellent Adherence Allowed Free Use of the Hand and Early Start of Exercise. Excellent Scarring Was Observed at 6 months (F).

**Figure 2.**
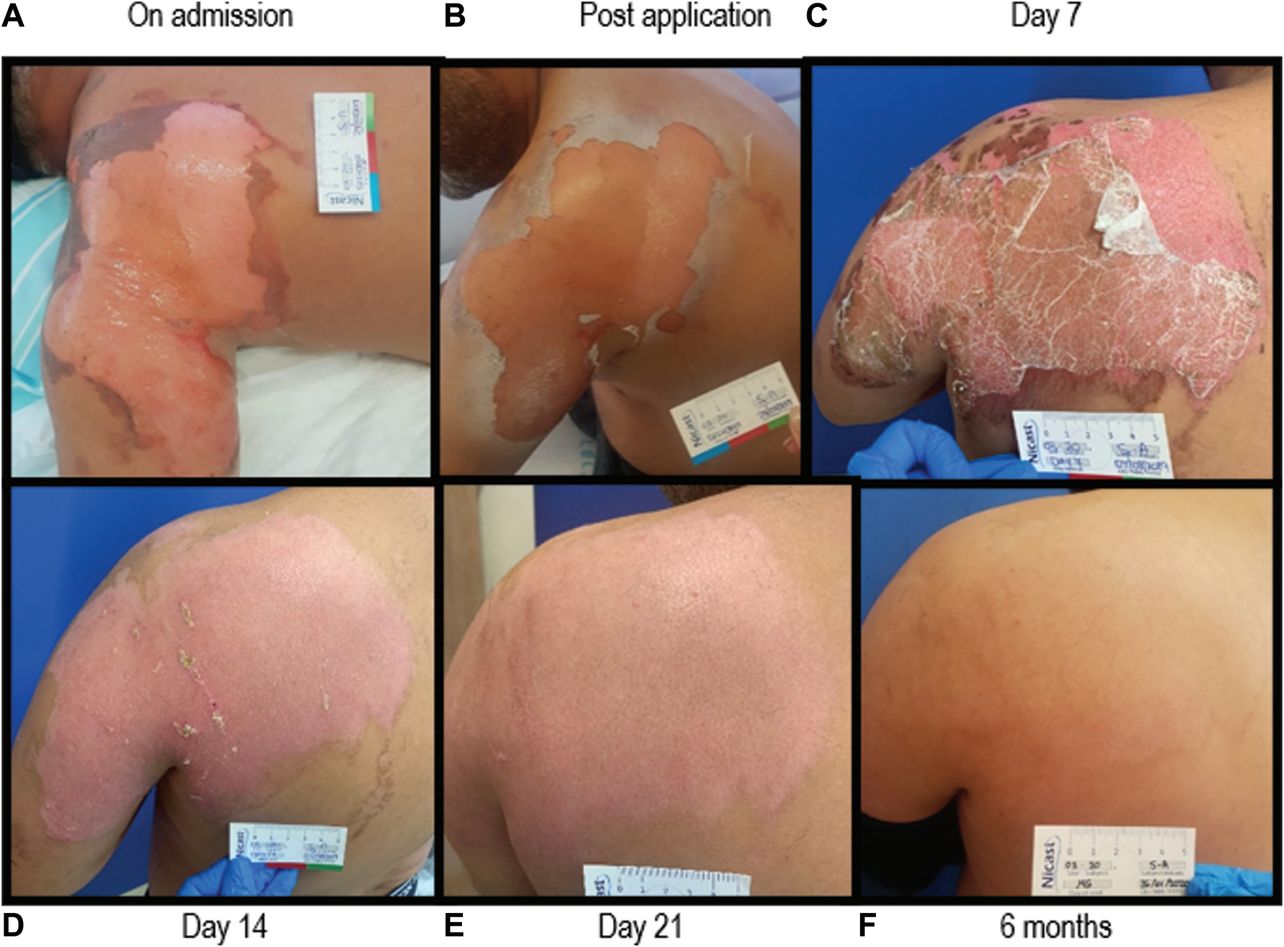
Forty-Two-Year-Old Male With Superficial Partial-Thickness Burn on the Left Shoulder, Proximal Arm, and Lower Back (A). A Spincare Matrix Was Applied on Admission and Posttreatment With a Hypochlorite Solution. Spincare Was Easily Applied to This Overly Complicated Area to Dress. Excellent Adherence Allowed the Immediate Start of Physiotherapy and Showers. Healing Progress on Day 7 (B) and Complete Healing by Days 14 and 21 (C, D) With Excellent Scarring at 6 months (E).

**Figure 3.**
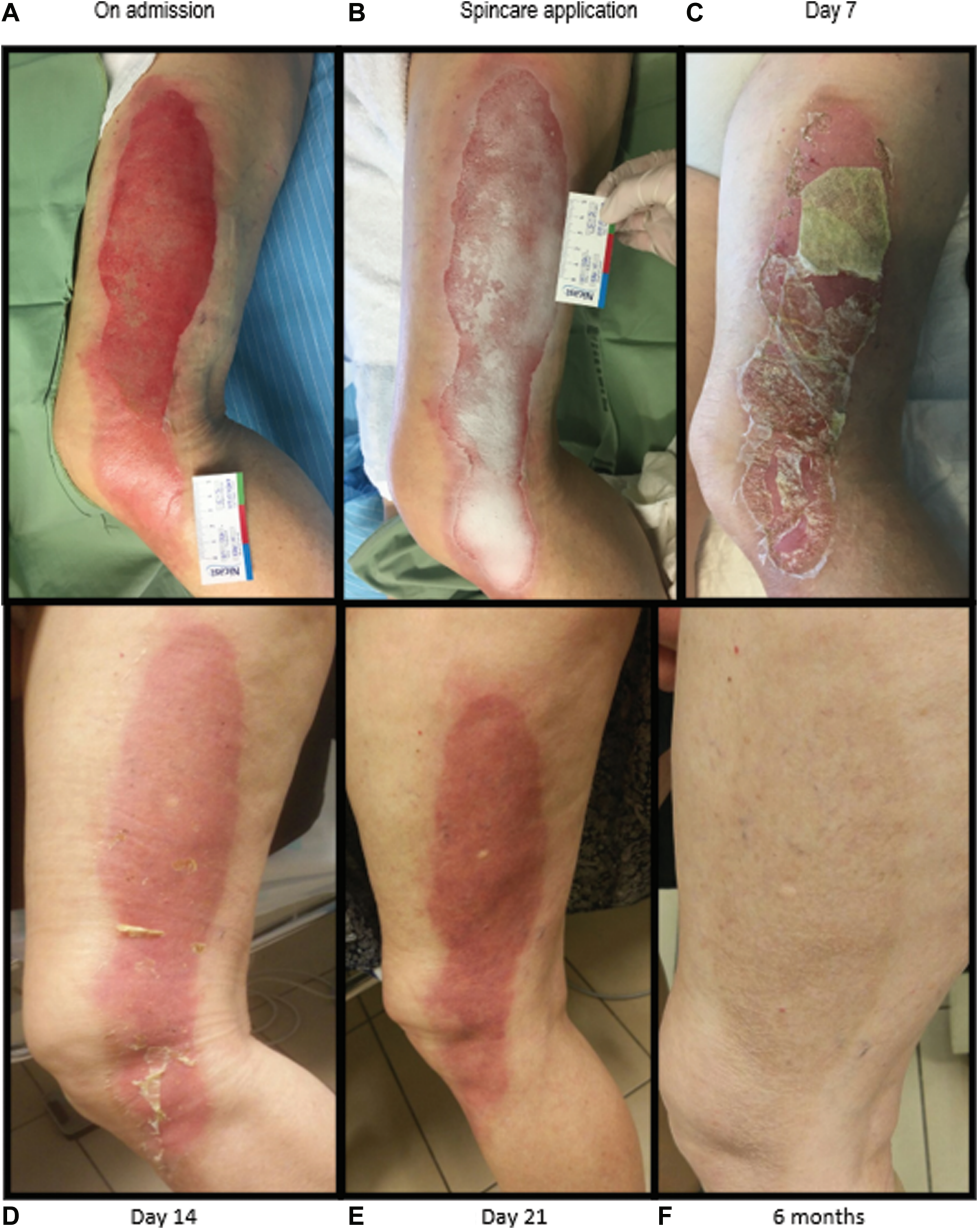
A 60-Year-Old Female With Partial-Thickness Burns Hot Water in the Lateral Area of the Left Thigh (A). Spincare Applied on Admission (B). Healing Progress on Day 7 (C) and Complete Healing by Days 14 and 21 (D, E) With Excellent Scarring at 6 months (F).

The mean healing time was 12.8 ± 4.3 days, and examples of typical healing processes can be seen in Figures 1–3.

### Primary outcome—safety

Dermal safety was assessed by Draize score: 0–4 scale for erythema and 0–4 scale for edema, and results are shown in Table 3. On day 7 assessment, most of the wounds were evaluated with no edema (32/41, 78.0%) and no erythema (29/41, 70.7%). On day 14, 26 of 36 wounds (72.2%) had no edema, and 19 of 36 wounds had no erythema (52.8%). On day 21, 32/36 (88.9%) of the wounds were evaluated with no edema, and 24/36 (66.7%) had no erythema. Those wounds with a Draize score > 0 were evaluated only with “Very slight edema” (score 1) on days 7, 14, and 21, and the highest erythema score recorded was 2.

**Table 3.** Evaluation of Dermal Safety in Wounds Treated With the Spincare System

Assessment	Follow up Day 7		Follow up Day 14		Follow up Day 21	
	*N*	%	*N*	%	*N*	%
Wounds	41	–	36	–	36	–
Erythema						
0 No Erythema	29	70.7	19	52.8	24	66.7
1 Very slight	11	26.8	12	33.3	7	19.4
2 Well defined	1	2.4	5	13.9	5	13.9
Edema						
0 No Edema	32	78.0	26	72.2	32	88.9
1 Very slight	9	22.0	10	27.8	4	11.1

### Secondary outcome—pain

The pain was assessed using the VAS from 1 to 10, where 0 indicates no pain and 10 indicates the worst pain experienced. The results are shown in Table 4. On procedure day 1, the mean VAS score during the procedure was 2.94 ± 2.93, compared to 2.50 ± 3.02 and 1.27 ± 2.07, pre- and postprocedure, respectively. On redressing visits, the VAS score was 4.53 ± 3.87 during the procedure, with a pain score of 3.00 ± 3.38 postprocedure (Table 4).

**Table 4.** Pain Assessment Pre, During, and Post-procedure using the Spincare System

	Procedure day (Day 1)			Re-dressing			All applications		
	*N* (wounds)	Mean	SD	*N* (wounds)	Mean	SD	*N* (wounds)	Mean	SD
Pain level—pre-procedure	52	2.50	3.02	15	3.67	3.48	67	2.76	3.14
Pain level—during the procedure	51	2.94	2.93	15	4.53	3.87	66	3.30	3.21
Pain level—post procedure	52	1.27	2.07	15	3.00	3.38	67	1.66	2.50

### Secondary outcome—itching

The itching was assessed based on a VAS from 1 to 10, where 10 indicates the worst experience. Table 5 presents the itching scores assessed on days 7, 14, and 21 and follow-up months 3 and 6. The highest and the lowest mean itching scores were recorded on day 14 (3.78 ± 4.07) and month 6 (1.88 ± 2.88), respectively.

**Table 5.** Itching Assessment of Wounds Treated with the Spincare System

Itching assessment	*N* (wounds)	Mean	SD
Follow-up visit (day 7)	41	2.73	2.94
Follow-up visit (day 14)	36	3.78	4.07
Follow-up visit (day 21)	36	2.92	3.52
Extended follow-up visits (Month 3)	32	2.00	2.72
Extended follow-up visits (Month 6)	34	1.88	2.88

### Secondary outcome—scarring

The total Vancouver Scar score was calculated as the sum of the 4 variables scores assessed: vascularity, height/thickness, pliability, and pigmentation (can reach 13 when all variables are at max). The total score was at the lower value of the Vancouver Scar Scale for all wounds (2.13 ± 2.45 and 2.00 ± 2.34 at month 3 and month 6, respectively) and less for superficial than intermediate wounds (Table 6).

**Table 6.** Scarring Assessment of Wounds Treated with the Spincare System using the Vancouver Scar Scale

Total score	Intermediate			Superficial			All		
	*N* (wounds)	Mean	SD	*N* (wounds)	Mean	SD	*N* (wounds)	Mean	SD
Follow-up visits (Month 3)	22	2.64	2.65	10	1.30	1.57	32	2.13	2.45
Follow-up visits (Month 6)	24	2.19	2.45	10	1.60	1.78	34	2.00	2.34

### Secondary outcome—AE

A summary of AE is presented in Table 7. A total of 17 AEs occurred in 13 out of 31 patients; one had 2 AEs. Of 17 AEs, 15 (88%) were mild, 1 moderate, and 1 severe. Fifteen AEs, including the severe one, were assessed as unrelated to the treatment. Furthermore, one minor occurrence (pain at the site of application) was determined to be “probably unrelated,” and one moderate adverse event—hypertrophic scar—was deemed to be “possibly related.” At the time of assessment, we were aware that this hypertrophic scar could potentially be a keloid. However, it was still challenging to differentiate between the two.

**Table 7.** Summary of Adverse Events by SOC and PT Recorded in Patients Treated with the Spincare System

MedDRA System Organ Class (SOC)	Preferred term (PT)	Severity	Relatedness	Subjects		Events	
				*N*	%	*N*	%
Ear and labyrinth disorders	Vertigo	Mild	Not related	1	2.3	1	5.9
Gastrointestinal disorders	Constipation	Mild	Not related	1	2.3	1	5.9
General disorders and administration site conditions	Application site pain	Mild	Probably not related	1	2.3	1	5.9
	Pyrexia	Mild	Not related	1	2.3	1	5.9
	Secretion discharge	Mild	Not related	1	2.3	1	5.9
Infections and infestations	Conjunctivitis	Mild	Not related	1	2.3	1	5.9
	Impetigo	Severe	Not related	1	2.3	1	5.9
	Infection	Mild	Not related	1	2.3	1	5.9
Nervous system disorders	Dizziness	Mild	Not related	1	2.3	1	5.9
	Syncope	Mild	Not related	1	2.3	1	5.9
Psychiatric disorders	Anxiety	Mild	Not related	1	2.3	1	5.9
	Poor quality sleep	Mild	Not related	1	2.3	1	5.9
Skin and subcutaneous tissue disorders	Keloid scar	Moderate	Related	1	2.3	1	5.9
	Pruritus	Mild	Not related	2	4.5	2	11.8
Vascular disorders	Bloody discharge	Mild	Not related	1	2.3	1	5.9
	Hypertension	Mild	Not related	1	2.3	1	5.9

### Secondary outcome—ease of use and time of dressing

In most wounds, the Spincare matrix was applied once and left on the wound throughout the healing process. In those cases, the matrix peels off on its own once healing is complete underneath. Thirteen subjects underwent redressing in addition to the first treatment; 3 of them underwent redressing twice, that is, a total of 16 redressing cases were recorded for 14 wounds, representing 27% occurrence of redressing.

The total number of 68 dressing applications to our patients using the Spincare System was evaluated, and a vast majority of them (~84%) reported it as “easy to apply”; only ~10% found the application of dressing by the Spincare System “moderate” or “hard to apply” (Table 8). Accordingly, 77% of the treatments could apply the matrix using the Spincare System in less than 10 min and 97% in less than 20 min. It took more than 20 min for only 3% of dressing applications (Table 8).

**Table 8.** Assessment of the Ease of Use of the Spincare System

Ease of use			Time to completion		
	*N*	%		*N*	%
Not done	1	1.47	NA	1	1.47
Easy to apply	57	83.82	Less than 5 min	24	35.29
Adequate	3	4.41	5–10 min	28	41.18
Moderate	6	8.82	11–20 min	13	19.12
Hard to apply	1	1.47	More than 20 min	2	2.94
**Total**	**68**	**100.00**		**68**	**100.00**

## DISCUSSION

This study evaluated the use of the Spincare System in managing partial-thickness burns and demonstrated effectiveness and safety with a sound healing process, healing time, and long-term results with high-quality scarring without complications. The study was observational, without internal controls, where all patients received dressing by the Spincare System. As such, we discuss our results in the context of published work investigating the use of other comparable dressings in partial-thickness burn management.

In their 4-year experience, Blome-Eberwein et al assessed healing outcomes and complications using an absorbable synthetic membrane (a synthetic skin substitute), Suprathel, to treat partial-thickness burns.^11^ The authors reported that the average time to heal was 13.7 days for ≥90% epithelialization, with 11.9 days for pediatric patients and 14.7 days for adults. In addition, the average pain level was 1.9 (2.7 for adults) throughout the treatment period on a 10-point scale. Applying the same healing criteria (≥90%) to our data (adult patients) indicated a shorter average healing time of 11.3 ± 3.8 days for our adult population treated with the Spincare System, indicating a shorter average healing time of approximately 3 days, which is on par with the healing of pediatric patients reported by Blome-Eberwein et al.^11^ Overall, the pain level associated with the Spincare System treatment was relatively low, with our study demonstrating lower pain levels measured during procedure days and even follow-up visits. These results indicated that the Spincare System might improve the healing process in adult patients. Substantially, infection is a common and significant risk factor in burn healing; only a single case of infection (~2% of the cohort) was noted in our patients, which, in addition, was unrelated to the Spincare matrix. Blome-Eberwein et al reported a more than 4 times higher proportion of infections (8.79%) in their adult cohort of patients.^11^

Another study by Highton et al examined Suprathel for partial-thickness burns in children and found that ~70% of their patients were healed at 21 days postinjury,^12^ much less than our results in adults presented here using the Spincare System (~92%). The authors also reported that the healing time of superficial partial-thickness and mid-dermal burns was similar,^12^ aligns with our results.

Franck David et al compared the performance of a soft silicone-coated wound contact layer (Mepitel One) with a lipid-colloid dressing (UrgoTul) in treating acute wounds in adult patients.^13^ These patients presented with an acute wound of traumatic origin (dermabrasion, skin tears, other) or a benign superficial thermal burn (less than 10% of total body surface) requiring dressings. The wound size was between 3 and 240 cm^2^. The authors reported a complete wound healing (100% re-epithelialization) by day 21 in ~66% of patients (39/59 patients) in the Mepitel group and ~44% of patients (27/62 patients) in the UrgoTul group.^13^ In this context, our data showed 81% healing (31/38 wounds) when applied the same criterion (100% re-epithelialization by day 21). Overall, the Spincare System provides comparable or better support for short-term healing processes than other commercially available dressing options for treating partial-thickness burns.

Another essential but long-term part of the healing process is scar formation, sometimes in the form of a thick raised keloid scar, indicating dysfunction of the wound healing process due to the overproduction of fibrinogen and collagen.^14–16^ Some significant risk factors for keloid formation include age under 30 and family history.^17^ It is important to note that a keloid differs from a hypertrophic scar.^15^ If a tendency to form keloids is known, some preventive steps could be taken, such as applying a pressure pad or a silicone gel pad for 6 months after a skin injury, though this does not represent a standard of care for burn injuries.^18^ In our study, only one patient (~2% of the total cohort) treated with the Spincare System matrix presented with a keloid formation, as opposed to ~14% of patients who received topical treatment with the Suprathel membrane as reported by Blome-Eberwein et al.^11^ Our one case of keloid formation was also reported as the only adverse event possibly associated with the Spincare matrix, indicating good safety properties of the system. In addition, most of our clinical personnel found the Spincare System easy to use and could complete the dressing in less than 10 min. This provides much better comfort to patients and shorter exposure of wounds to the environment, reflected in relatively low pain levels and minimal infections.

This study demonstrated the advantages of Spincare beyond the study endpoints worth addressing. The matrix showed excellent adherence on all wounds, even in hard-to-heal areas such as hands and shoulders; this potentially makes the dressing phase much quicker and easier for the hospital team. The matrix remained on the wound through the healing period and peeled off when full epithelization was achieved. This, in turn, allowed us to avoid secondary dressing in a very early stage, which sequentially allowed the freedom to move, maintenance of a range of motion and no loss of function, early start of physiotherapy and regular showers, potentially contributing to patients’ quality of life in this challenging period.

Finally, our study was conducted in a real-world clinical environment. As such, this design inherently includes a limitation, as no internal control was involved. However, we were able to compare our results with already published work evaluating comparable treatments in comparable clinical spaces. The Spincare System performed well in this comparison, showing better results than comparable studies. Our results warrant a more extensive study in the framework of the controlled conditions of a clinical trial.

## CONCLUSION

Current findings indicate that using the Spincare System in managing partial-thickness burns is safe and effective while suggesting advantages such as excellent adherence, ease of use in a hard-to-dress area, early removal of secondary dressings, and early showers. Spincare achieved rapid and complete wound healing with a low incidence of AE.

## Notes

*Author Contributions:* Josef Haik (Conceptualization [Lead]; Investigation [Equal]; Resources [Equal]; Validation [Equal]; Visualization [Equal]; Writing—original draft [Equal]; Writing—review & editing [Equal]), Yehuda Ullmann (Data curation [Equal]; Investigation [Equal]; Project administration [Equal]; Supervision [Equal]; Validation: Supporting; Visualization: Supporting; Writing—review & editing: Supporting), Eyal Gur (Data curation [Equal]; Investigation [Equal]; Project administration [Equal]; Supervision [Equal]; Validation: Supporting; Visualization: Supporting; Writing—review & editing: Supporting), Erik Biros (Data curation: Supporting; Formal analysis [Equal]; Validation: Supporting; Visualization: Supporting; Writing—original draft: Supporting; Writin—review & editing: Supporting), Rachel Anne Kornhaber (Validation: Supporting; Visualization: Supporting; Writing—original draft: Supporting; Writing—review & editing: Supporting), Michelle Cleary (Validation: Supporting; Visualization: Supporting; Writing—original draft: Supporting; Writing—review & editing: Supporting), Dani Kruchevsky (Data curation [Equal]; Formal analysis [Equal]; Investigation [Equal]; Validation: Supporting; Visualization: Supporting; Writing—review & editing: Supporting), Sivan Zissman (Validation: Supporting; Visualization: Supporting; Writing—review & editing: Supporting), Yossi Namir (Validation: Supporting; Visualization: Supporting; Writing—review & editing: Supporting), Moti Harats (Conceptualization [Equal]; Data curation [Equal]; Formal analysis [Equal]; Investigation [Equal]; Methodology [Equal]; Project administration [Lead]; Supervision [Lead]; Validation [Equal]; Visualization [Equal]; Writing—original draft [Equal]; Writing—review & editing [Equal])

*Funding:* This study is part of a project sponsored by the European Commission H2020 SME Grant 671970 to Nanomedic Technologies Ltd. (a subsidiary of Nicast trial).
